# Assessing the health-related management of people with differences of sex development

**DOI:** 10.1007/s12020-021-02627-y

**Published:** 2021-01-30

**Authors:** Martina Jürgensen, Marion Rapp, Ulla Döhnert, Fabian-Simon Frielitz, Faisal Ahmed, Martine Cools, Ute Thyen, Olaf Hiort

**Affiliations:** 1 Division of Paediatric Endocrinology and Diabetes, Department of Paediatrics and Adolescent Medicine, University of Lübeck, Lübeck, Germany; 2 Institute for Social Medicine and Epidemiology, University of Lübeck, Lübeck, Germany; 3 Office for Rare Conditions, University of Glasgow, Glasgow, UK; 4 Department of Medicine, Division of Endocrinology and Centre for Endocrine Tumors, Leiden University Medical Centre, Leiden, The Netherlands; 5 Division of Paediatric Endocrinology, Department of Paediatrics and Internal Medicine, Ghent University Hospital, Ghent University, Ghent, Belgium; 6 Department of Paediatrics and Adolescent Medicine, University of Lübeck, Lübeck, Germany

**Keywords:** Differences of sex development, Health care system, Nonbinary concept of sex, Quality indicator, Benchmark

## Abstract

**Purpose:**

Health care requirements and perception of people with differences of sex development (DSD) have changed enormously since the “Chicago Consensus Conference” in 2005. Therefore, new standards of care and evaluation of care have to be developed.

**Methods:**

We summarize the social and legal approach to care for DSD during the last two decades and report the main results of European research activities.

**Results:**

The last two decades were accompanied by legal and societal discussion regarding how to deal with a nonbinary concept of sex. This leads to the necessity to assess health care requirements for individuals with DSD in an objective manner. We briefly review the results of the recently funded European research projects dealing with health-related issues in DSD like EU COST Action DSD, I-DSD, and dsd-LIFE, and address the compilation of quality indicators that will be needed to benchmark health care provision and health care-related outcomes.

**Conclusions:**

The benchmarking process has to be implemented among health care providers for individuals with DSD within the European Reference Networks for Rare Conditions.

## Introduction

Differences/disorders of sex development (DSD) describe congenital conditions affecting sex development with a broad spectrum of clinical, genetic, and laboratory findings [1]. In the last decades, DSDs have undergone enormous changes in general and medical perception, as well as societal acceptance and medical care [2]. In this paper, we will outline, how the increasing demand from people with DSD, but also from health care professionals and the general public has changed established clinical practices for the management of patients within the health care systems [3, 4]. In 2005, an international consensus conference was held, which received wide acceptance for its change in nomenclature and advice for management of the conditions [1]. This new terminology and classification have given a scientific basis for biological perception and have promoted research both in the pathophysiological understanding as well as in health services research for DSD. At the same time discussions with patient organizations and public debate have triggered claims for further actions that could enhance acceptance of the conditions and decrease societal taboo.

One reason for the ongoing discussion about how to deal with DSD could be that “sex/gender” is not just one of many equal value categories, but rather one that is of particular relevance not only for the individual but also for society [5]. At the individual level, sex/gender is one of the key elements of identity, self-image and sexuality and potential fertility. In relation to all of these very intimate aspects of personality, humans are extremely sensitive and vulnerable. In addition to the individual meaning, gender role, sexuality, and reproduction are aspects of human beings that are subject to strong social regulation in all cultures and times [6]. This is one of the classic areas of negotiation between individual self-realization and personal freedom, as well as social norms and controls.

Nowadays the initially used term “disorder of sex development” has been exchanged by “differences of sex development”, in order to challenge the traditionally binary concept of sex and avoid socially inappropriate language [7]. The negative notion of disorder has been changed to the neutral term differences to strengthen and empower people to live with the social and societal aspects of DSD. Nevertheless, people with DSD will need entry into the medical system for various reasons including confirmation of diagnosis, thorough information about their condition as well as appropriate care of eventual medical and psychosocial consequences of the conditions [4, 8, 9]. At this time, we have to assess the needs of patients with DSD in our health care systems, benchmark the quality of care that is delivered at specialized centers of care, and analyze effects on patient care and health-related quality of life.

## Needs for changing health care in DSD

DSD is an umbrella term for a large number of conditions that affect sex development; they are classified as “rare diseases” [1, 2, 10]. From a medical point of view only few forms of DSD require acute medical action. Nevertheless, there is a need for medical treatment in many respects, which, however, can vary greatly depending on the diagnosis, age, and personal needs [3]. First and foremost, medicine has the task of confirming a diagnosis and analyzing possible consequences of the variant gender development (e.g., comorbidities, hormonal peculiarities). Other areas in which medicine plays an important role for people with DSD are pubertal development and sexual maturation, mental health, sexual health, and fertility [3, 11].

Addressing the medical needs of people with DSD has long been characterized by adjusting the individual to the traditionally binary social sex/ gender norm (also surgically) [1, 5, 12]. Over the last 50 years, this approach has been increasingly criticized, when DSD-advocacy groups denounced the poor outcomes of medical and psychological care and demanded the right of intersex people to self-determination, bodily integrity, and protection from discrimination [13, 14]. In particular, children’s rights—and here the right to an open future, in which they can later determine their own gender and the associated protection against “cosmetic” interventions—came into focus [15, 16]. The demands of these groups can be found in the context of other empowerment movements, which, for example, advocate the enforcement of patient rights (shared decision making, informed consent, patient centered outcomes, e.g.), and are based on the implementation of child and minority rights [17, 18].

As a consequence, a change of perspective has occurred in some legal systems. In the European Union DSD issues have progressively emerged as being relevant to fundamental rights protection. For example, in 2012 the German Ethics Council (Ethikrat) published a comprehensive opinion on intersex issues, providing a range of recommendations to safeguard the rights of intersex people [19]. A challenge in changing legal frameworks is to consider the latest ethical, psychological, sociological, and medical findings wherever possible and to reflect each case on its own merits [20]. In view of the complexity, the changes at the legal level were implemented as an amendment to the German Civil Status Act in December 2018 [21]. In addition to the previous binary or non-registration of the sex entry, the act now also allows people with DSD in Germany to use the third category “diverse” instead of “male” and “female” and provides for facilitating regulations for changes in the birth register (§§ 45 b, 22 para. 3 PStG). The “diverse” category can also be applied in adult people with DSD. In the meantime, there have also been changes in many other countries of the EU and outside. In February 2019, the European Parliament adopted a landmark resolution on the “Rights of Intersex People” [18]. Thereby the European Parliament sets a clear standard within the European Union for the protection of intersex people’s body integrity and human rights. The resolution complements the 2017 resolution “Promoting the human rights of and eliminating discrimination against intersex people” adopted by the Parliamentary Assembly of the Council of Europe [22].

## European research activities

Following the 2005 Consensus Workshop that stressed the need for the regular collection and sharing of data across geographical boundaries, a concerted effort through funding from the European Society for Paediatric Endocrinology, an EUFP7 grant and subsequently the MRC UK, led to the development of the ESPE DSD Registry, which was succeeded by the European DSD Registry and subsequently the I-DSD Registry [23]. With allied projects such as the EU COST Action DSDnet, the FP7 project DSD-Life, the I-DSD Registry and its allied registry, I-CAH, has not only supported research activities but also paved the way to the benchmarking of services [3, 24]. Audit of clinical activity, including patient reported experience measures, participating in disease registries, building collaborative working partnerships, attendance at joint clinics and education events are crucial if knowledge and information sharing is to be optimized across clinical teams [25]. There is a need for dedicated time for overseeing and/or performing these tasks that allow structured management as well as audit of a complex service, which would exemplify the center’s commitment to quality assurance [8]. In this context, a clear example where joint collaborative activities have the potential to lead to an improvement in care quality is the model of the European Reference Networks (ERN) that have been funded by the EC for improving the care of people with rare conditions [26]. There are at least two ERNs, Endo-ERN and eUROGEN, which consist of reference centers for managing the care of people with conditions associated with DSD. The challenge for these ERNs is to show that their creation has led to an improvement in care and for this, there is a clear need to measure and compare easily collected indicators of care quality through projects such as EuRRECa [27].

## Measuring the quality of health care: development of quality indicators for the care of people with DSD

Health care needs assessment is based on three elements: (1) existence of a nontrivial health risk or disorder (e.g., to be graded on severity and prognosis); (2) potential availability of, and access to services, and (3) patient’s ability to benefit from services [28]. In DSD, the ability to benefit from health care services is less well studied and there is scarce evidence on the efficacy of therapies which is also due to the difficulty of studying an extremely heterogenous and in the specific diagnoses rare condition [1, 2].

The subjective and objective appraisal of the quality of care people receive includes measurement of satisfaction with services [9, 29]. Variations in treatment processes are expected to be associated with patient reported outcomes (PRO) related to patients’ satisfaction with care. Describing components of care requires their systematic identification and operationalization.

In general, quality indicators address relevant and measurable aspects of care and indirectly depict the quality of a unit through numbers or numerical ratios [28]. Evaluation of health care quality in DSD is difficult, because there is no gold standard for guideline-compliant and quality-assured treatment processes and structural components of health care institutions. To develop quality indicators, one relies on the Donabedian model, with structure, process, and result quality as central, interdependent quality dimensions in health care [30]. Structural quality is defined as the description of the framework conditions such as equipment, availability and quality of laboratories, qualification of personnel, formal training of teams, physical accessibility for patients and other aspects that assess structural attributes of the institution. The quality of the process relates to the way in which the service is provided and describes all activities that are carried out in the course of the individual care in DSD, e.g., multiprofessional conferences, shared decision making, quality of physician patient communication, waiting times, patient orientation, the process of obtainment of informed consent, or the delivery of therapies such as hormonal or fertility treatment. The quality of the process is usually assessed from the patients’ perspective, i.e., measuring satisfaction with care [31]. The quality of the results focusses on treatment outcomes collected through subjective assessment of the patient (e.g., quality of life) and medical data, such as complication rates and standardized evaluation of functionality. To ensure good structural or process quality in DSD, the starting point in developing quality indicators is to research the available national and international guidelines and operationalize the recommendations identified there [28, 32]. In addition, an extensive literature search is necessary to identify further aspects that are named as decisive for good care in DSD. Finally, background information and references to other relevant aspects with regard to quality of care have to be collected through qualitative interviews with relevant stakeholders in the field of DSD, such as medical expert team members, affected individuals and self-organizations.

Indicators for the structural and process quality of care must meet main criteria: relevance, sensitivity, specificity, reliability, operational feasibility, and practicability [32]. A first pilot assessment is used to check an initial set of indicators with regard to feasibility and performance of each indicator. Static analyses of floor and ceiling effects are carried out and “underperforming” indicators are eliminated. Well-performing indicators build the set of quality indicators for further assessment. National guidelines and expert opinions may influence the chosen set of quality indicators, which can therefore differ between countries.

## Examples of measuring the quality of care: assessing the needs for health care of people with DSD

Among standardized instruments to assess satisfaction with care, the international customer satisfaction questionnaire (CSQ-8) [33] and the Child/Youth Health Care-Satisfaction, Utilization and Needs (C/YHC-SUN) [34, 35] have been used in the population with DSD. The CSQ-8 is a self-report questionnaire with eight items constructed to measure satisfaction with services in general [33], of which two shortened versions (CSQ-3, CSQ-4) exist [36]. Primarily for children and adolescents with chronic conditions, the long and short version of the CHC-SUN [34] and the YHC-SUN [35] were developed and later adapted for adults [29]. The instruments consist of items in respect to provision, utilization, access problems, and satisfaction with general practitioner, specialist care, prescribed medicine, and emergency services. Furthermore, unmet needs are assessed in 16 services and can be adapted due to the evaluated health care setting. Further items are related to satisfaction with care in the last 12 months and comprise six domains “diagnosis/information”, “coordination”, “child-(patient-)-centered care”, “hospital (clinic) environment”, “doctors’ behavior”, and “school related services” as well as a single item on “general satisfaction with health care”.

One national study, called German network study [37], and one European study, dsd-LIFE, [38] evaluated the needs for health care of people with DSD. In the German network study between 2005 and 2007, a total of 439 children, adolescents and adults with DSD were asked about their satisfaction with specialist care [37]. In adults, the greatest satisfaction with the specialist care was reported by women with CAH, the lowest by participants with 46,XY DSD and very rare DSDs [9]. In children, parents of girls with CAH also reported greater satisfaction with specialist treatment compared to other diagnostic groups. Satisfaction was lowest among parents of children with 46,XY DSD who grew up as a male; in particular, dissatisfaction with the diagnostic process was high [39]. Regarding unmet needs, the need for psychosocial support was reported by more than half of the parents with children with 46,XY DSD; but only half who stated a need had received a corresponding psychosocial care [40]. More than one decade ago, this national study demonstrated the need for improvement in the diagnostic process and for inclusion of psychosocial care into the treatment of people with DSD.

In the European study dsd-LIFE, 1040 young people and adults with DSD as described in the classification system of the Chicago Consensus Conference took part in 2014/2015 [38]. Dsd-LIFE consisted of two study parts, one included a medical interview, a retrospective chart review and medical examinations following standard operation procedures, the other one a PRO questionnaire. The PRO included sociodemographic data standardized questionnaires about the general quality of life, psychological well-being, psychosexual development, sexuality and condition specific self-constructed items as well as satisfaction with health care (CSQ-4; YHC-SUN-SF) [33, 35]. Within the European study, a center score was developed for the level of multidisciplinary care in each center for each diagnosis group based on the criteria of the European Reference Network on Rare Endocrine Conditions (Endo-ERN) [29]. This score comprised the infrastructure at the hospital level (size of the center, acknowledgment as a center of reference, participation in registries, national or international collaborations, participation in clinical trials, access to molecular genetic testing), infrastructure at the clinic level (team-size, options for referrals) and the service delivery (availability of case management, transitional care, collaboration with patient organizations, conduct of case conferences, access to educational materials for patients, telephone counseling). Participants with CAH and Turner syndrome reported a tendency toward higher satisfaction with health care with only small differences overall between the diagnostic groups. However, it was clearly shown that a higher level of multidisciplinary care in a center (higher “center score”) was associated with a higher level of satisfaction. This was true for all four diagnosis groups, but especially for all participants who self-reported a good to very good health status. This finding is even more important because across all conditions, health status explained most of the variance in quality of life [41]. In addition, this European study revealed the need to decrease variability between centers of reference/competence, especially in endocrine and fertility treatment [11, 42]. The impact of care should not be assessed only on patient satisfaction with care but also on patient health outcomes like the general health status, which is often not related to the condition itself but classified as co-morbidity [43] or the mental health [44]. These findings have major impact on the organization of care. Individuals with DSD need both highly specialized care as well as a coordinating medical center, addressing all issues of health and collaborating with subspecialists.

## Discussion and outlook

Research aiming to maintain or improve people’s health includes basic science research, clinical studies evaluating the effect of novel medical interventions on health outcomes, including body structures and functions, physical and mental performance, and quality of life. However, health services research includes determinants other than individual physical and mental health characteristics but environmental factors such as the health care patients receive. This is important to study because these determinants are amendable to change and improvement in quality of care may result in better health. Beyond quantitative empirical data on the association of aspects of structural and process quality, projects should include qualitative empirical data from both interviews with individuals and group discussions (focus groups). Meaningful interpretation of empirical data must be developed jointly with people affected by DSD. Any project embarking on the study of quality of care should develop a participatory research design including a wide variety of age groups and clinical conditions. The emerging advocacy groups and self-organizations have been an important source to clarify the importance and appropriateness of measurements.
